# Arbitrary Symmetric Running Gait Generation for an Underactuated Biped Model

**DOI:** 10.1371/journal.pone.0170122

**Published:** 2017-01-24

**Authors:** Behnam Dadashzadeh, Mohammad Esmaeili, Chris Macnab

**Affiliations:** 1 Department of Mechatronics Engineering, School of Engineering Emerging Technologies, University of Tabriz, Tabriz, Iran; 2 Department of Electrical and Computer Engineering, University of Calgary, Calgary, Canada; University e-Campus, ITALY

## Abstract

This paper investigates generating symmetric trajectories for an underactuated biped during the stance phase of running. We use a point mass biped (PMB) model for gait analysis that consists of a prismatic force actuator on a massless leg. The significance of this model is its ability to generate more general and versatile running gaits than the spring-loaded inverted pendulum (SLIP) model, making it more suitable as a template for real robots. The algorithm plans the necessary leg actuator force to cause the robot center of mass to undergo arbitrary trajectories in stance with any arbitrary attack angle and velocity angle. The necessary actuator forces follow from the inverse kinematics and dynamics. Then these calculated forces become the control input to the dynamic model. We compare various center-of-mass trajectories, including a circular arc and polynomials of the degrees 2, 4 and 6. The cost of transport and maximum leg force are calculated for various attack angles and velocity angles. The results show that choosing the velocity angle as small as possible is beneficial, but the angle of attack has an optimum value. We also find a new result: there exist biped running gaits with double-hump ground reaction force profiles which result in less maximum leg force than single-hump profiles.

## Introduction

The field of legged locomotion has proposed some fundamental models, including the Spring Loaded Inverted Pendulum (SLIP), the active SLIP, and the Point Mass Biped (PMB). These models allow one to investigate both walking and running gaits. The SLIP model remains a popular tool to investigate bipedal walking and running gaits ‎[1]. This passive model, consisting of a point mass and a massless spring leg, can generate a center-of-mass (COM) trajectory and a ground-reaction-force (GRF) profile similar to that of human running ‎[2]. This has encouraged researchers to investigate SLIP to try to understand the fundamental dynamics of bipedal running ‎[3], and also to use it as a template for controlling real biped robots ‎[4]. For any forward velocity, SLIP can generate periodic running gaits that are passively stable in a narrow region of initial condition parameters, but that are unstable for parameters outside it ‎[5,6]. Therefore, some researchers have proposed stabilizing flight-phase and stance-phase controllers for SLIP running. Flight-phase controllers of the passive SLIP model prepare the swing leg for landing. One bio-inspired strategy uses swing-leg retraction, where the swing leg rotates backwards in the second half of the flight phase ‎[7,‎^8^]. A dead-beat controller can reject disturbances by adjusting the attack angle or spring stiffness ‎[9]. A control can update each attack angle to be the negative value of the previous take-off angle ‎[10]. Stance-phase controllers require an actuated SLIP architecture in order to add or remove energy from the system. Schmitt *et al* ‎[11] proposed a stabilizing control law for an active SLIP with a force actuator parallel to the spring. Seipel *et al* ‎[12] and Ankarali *et al* ‎[13] proposed control of an active SLIP with torque actuation at the hip and a spring-damper in the leg. Piovan and Byl ‎[14] considered an active SLIP model with a displacement actuator in series to the spring and presented a control strategy. (Note that these works add some control actions to the SLIP model in order to stabilize the natural gait).

As an alternative to SLIP, Srinivasan and Ruina ‎[15] proposed using a point mass biped (PMB) to investigate biped walking and running gaits using numerical optimization. The PMB consists of a point-foot, a point-mass as the body, and a prismatic-force actuator (instead of a spring) on a massless leg. The PMB is “perhaps the simplest mechanical model that is capable of exhibiting a broad range of gaits including walking and running” ‎[15]. They minimized the mechanical-work cost using numerical optimal control methods, obtaining piecewise linear functions that describe optimal walking and running leg-force profiles. They then described finite-force profiles that approximate the impulsive forces. Rebula and Kuo ‎[16] generated smoother and more human-like force profiles for the same model by introducing an adjustable weighting between work cost and force-like costs (including leg force and its *n*th time derivatives). They generated single-hump force profiles for optimal running gaits and double-hump profiles for optimal walking gaits. In this work, we also use PMB instead of SLIP, since it is a more general and fundamental biped model which allows one to investigate more general gaits. Because PMB is an active model not restricted to the trajectories and gait specifications imposed by the leg spring of a SLIP model, it provides a better template for real multibody active robots. For example, there is a unique attack angle for each specific forward velocity in SLIP periodic running, but not with a PMB model. Although both SLIP and PMB can generate human-like gaits (qualitatively speaking), in this work we aim to investigate the effects of choosing various analytic stance trajectories on gait optimality. With proper choice of parameters, we find these general analytic trajectories can also generate human-like gait dynamics. Our work is different from the previous works in ‎[15,‎^16^], since we can generate any desired trajectory using the same model and then choose the most optimal gait among these general trajectories. The selected general analytic trajectories are also able to generate human-like gait dynamics with proper choice of parameters.

Significant experimental biped robots include the Raibert hopper (a SLIP-like robot with pneumatic actuated legs) ‎[17], ASIMO (a zero-moment-point controlled walking and running robot with rigid actuation system) ‎[18], MABEL (a hybrid-zero-dynamics (HZD) walking and running robot with a parallel elastic actuation system) ‎[19], ATRIAS (a SLIP-based walking and running robot with series elastic actuation system) ‎[20], and PETMAN (a versatile walking robot developed by Boston Dynamics). However, achieving the agility and efficiency of human running remain open problems. One promising control strategy is to make the real robot follow a minimal model as a template ‎[4]. Poulakakis *et al* ‎[21] used SLIP as the HZD of an asymmetric hopper robot in order to generate stable planar hopping motion. Wensing *et al* ‎[22] used 3D SLIP as a template to control 3D running and steering of a humanoid robot model. Feedback linearization accomplished tracking in a SLIP COM trajectory of a three-link rigid hopper ‎[23]. Two active SLIP architectures with one and two degrees of actuation were proposed in ‎[24] as templates for ATRIAS running; the paper proposed a control strategy to follow SLIP, tested successfully in simulations. The results showed that motor torque saturation remains an obstacle to building practical biped running robots.

One aim of our work is to develop techniques that require less peak force for the stance leg. The success of the template-anchor methods described above motivates the approach in this paper where we use a more general and versatile template than the SLIP model. We propose a novel control law to make an underactuated robot’s COM follow any smooth trajectory with any arbitrary initial and final stance conditions (using the PMB model); this result should make it easier to control real biped robots subject to real-world constraints like motor torque saturation. (Note that biped robots with point feet are underactuated in stance phase making them uncontrollable using classic feedback control methods.) Here we compare a circular arc and polynomials of various degrees as COM trajectories in stance phase, but our methods can also be applied to other smooth analytical paths. The variation of cost-of-transport (COT), the maximum GRF with respect to angle of attack, and the COM velocity angle serve as criteria to evaluate the proposed methods.

## Biped Models

**Fig 1:**
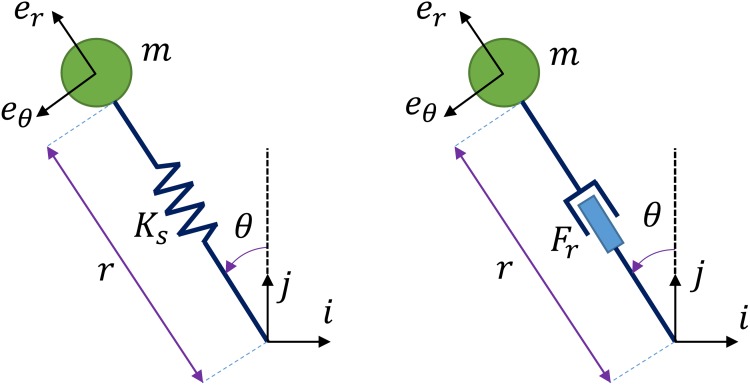
Parameters of (a) SLIP and (b) PMB model in stance phase.

The benefits of SLIP include its simplicity and its ability to capture some important dynamics of human running ‎[25]. However, it is totally passive. We propose to use PMB in this work in order to capture the advantages of SLIP while avoiding its passivity disadvantage, although we do compare our results to SLIP. PMB consists of a modified SLIP model where a force actuator replaces the spring (Fig 1). The stance leg in both models endures only compressive forces to keep the foot in contact with the ground. Unlike SLIP, PMB can generate arbitrary running gaits.

One step of a running gait includes a stance phase, a take-off, a flight phase and a touch-down. Stance and flight phases are continuous time phases, while take-off and touch-down are instantaneous events. In the stance phase the foot is assumed to be an ideal frictionless passive pivot to the ground. Take-off is an instantaneous transition from stance to flight phase with no discontinuities, occurring when the vertical component of the GRF becomes zero. In flight the robot behaves as a ballistic point mass. Due to the massless legs, there is no impact at touch-down and so no changes occur in the robot’s configuration and velocities. The touch-down state becomes the next stance phase initial condition, with no discontinuities.

In stance phase both SLIP and PMB models have 2 DOFs that can be modeled using Cartesian or polar coordinate systems. The body mass is denoted by *m*, the leg length by *r*, the leg angle with respect to vertical by *θ*, the leg axial force in PMB by *F*_*r*_, the spring stiffness in SLIP by *K*_*s*_, the Cartesian unit vectors by *i* and *j*, and the polar unit vectors by *e*_*r*_ and *e*_*θ*_. We assume stance state vector $x_{s}=\left[r,\;\theta ,\;\dot{r},\;\dot{\theta }\right]$. So $x_{s_{0}}=\left[r_{0},\;\theta_{0},\;{\dot{r}}_{0},\;{\dot{\theta }}_{0}\right]$ constitutes the initial condition of stance. The stance phase equations of motion for SLIP using Cartesian coordinates are

$$\left\{ \begin{array}{l} m\ddot{x}=K_{s}\left(r-r_{0}\right)\text{sin }\theta \\ m\ddot{y}=K_{s}\left(r_{0}-r\right)\text{cos }\theta -mg \end{array}\right.$$ (1)

which constitutes a passive system that utilizes elastic potential energy to boost the mass. On the other hand, PMB

$$\left\{ \begin{array}{l} m\ddot{x}=-F_{r}\text{ sin }\theta \\ m\ddot{y}=F_{r}\text{ cos }\theta -mg \end{array}\right.$$ (2)

is an active model without elastic elements and uses only control input *F*_*r*_ to boost the mass.

In flight, the ballistic motion of the robot can be described in a straightforward manner using Cartesian coordinates. The massless leg is assumed to be able to move to any desired angle instantaneously with no energy consumption. The flight state vector is $x_{f}=\left[x,\;y,\;\dot{x},\;\dot{y}\right]$. The dynamic model for running can be expressed as in the equation below, where + and - superscripts show the instances just after and just before events, respectively.

{x˙s=fs(xs,ts)xf+=Δfs(xs−)x˙f=ff(xf,tf)xs+=Δsf(xf−) (3)

## Running Gait Generation

For the stance phase trajectories we try a circular arc and polynomials of different degrees. Our method generates running gaits and calculates the necessary leg force profiles in stance phase. The proposed method will work for any desired stance phase path profile consistent with the initial condition.

### Circular-arc trajectory in stance phase

**Fig 2:**
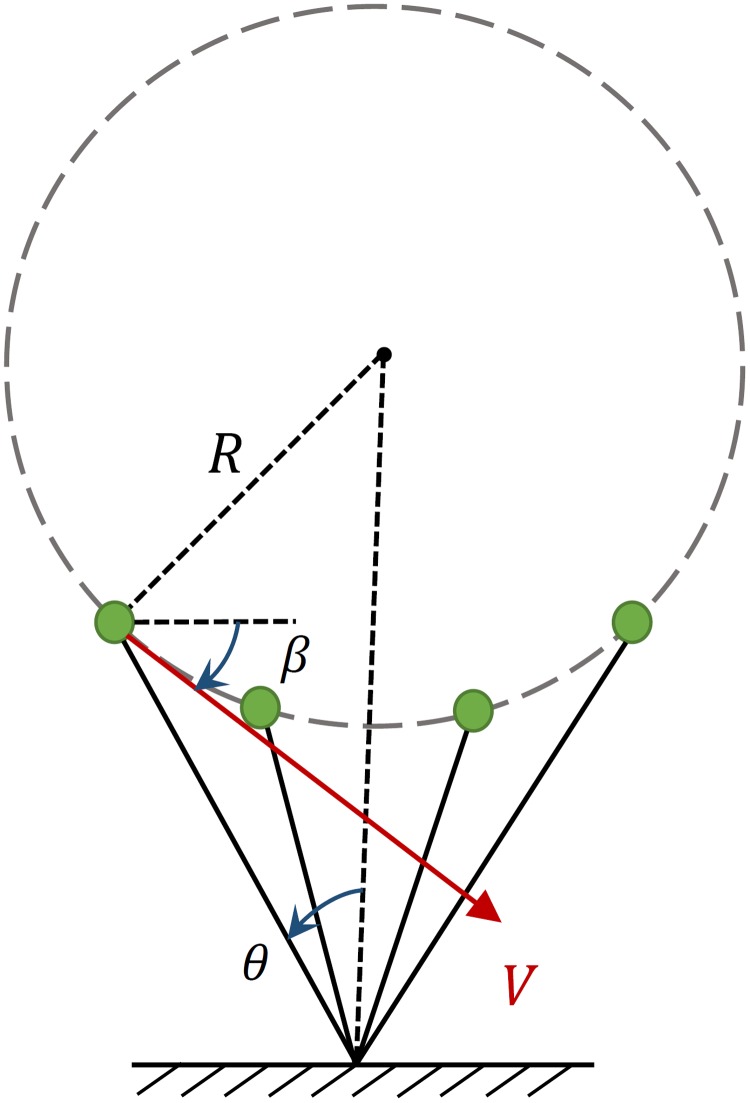
The conceptual circle trajectory and initial velocity vector for stance phase.

We desire a symmetric circular-arc trajectory to be generated using the single force actuator of the leg as shown in Fig 2. The center and the radius of the circle are defined using the touch-down angle, leg length and velocity vector direction. The circle is tangent to the initial velocity vector and its center is located at the intersection of the vertical line passing through the foot contact point and the radius line perpendicular to the initial velocity vector. So the stance phase trajectory becomes symmetric with initial and final positions mirrored with respect to the vertical, and initial and final speeds equal to each other.

Using a polar coordinate system with the origin at foot contact point, the velocity of COM is

$$\vec{v}=\dot{r}{\vec{e}}_{r}+r\dot{\theta }{\vec{e}}_{\theta }$$ (4)

with *r* and θ defined in Fig 1. This equation is stated in Cartesian coordinates as

$$\vec{v}=(-\dot{r}\text{ sin }\theta -r\dot{\theta }\text{ cos }\theta )\vec{i}+(\dot{r}\text{ cos }\theta -r\dot{\theta }\text{ sin }\theta )\vec{j}.$$ (5)

**Fig 3:**
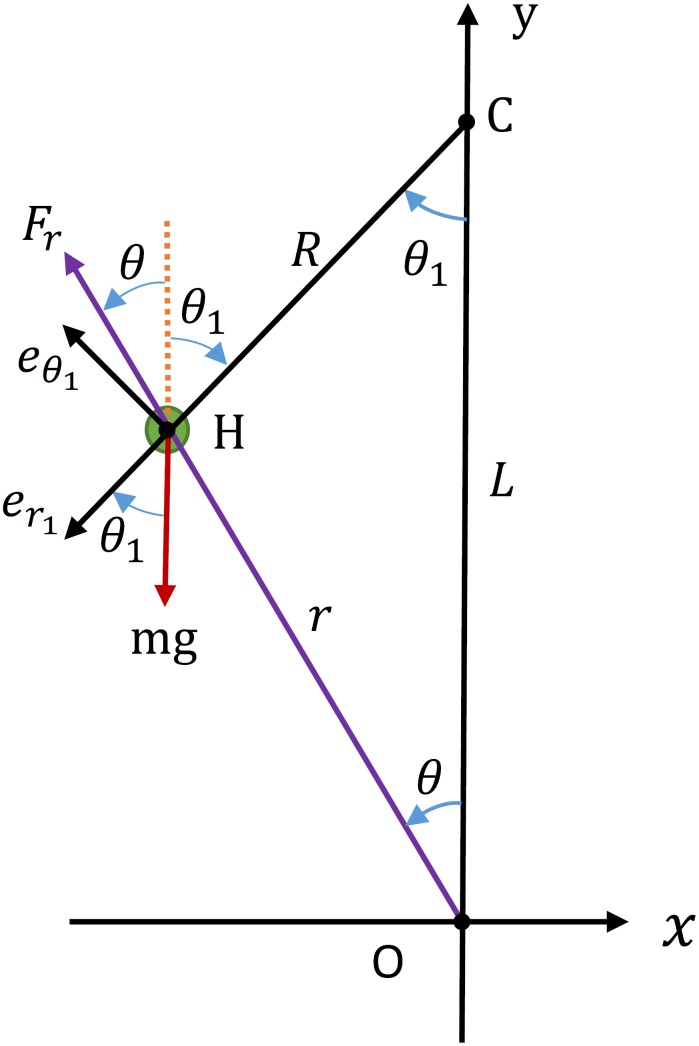
Free body diagram of PMB on circular-arc trajectory.

Fig 3 shows the free body diagram of PMB on a circular-arc trajectory. In this figure point O is the foot contact point, point H is the hip point with point mass *m* located on, and point C is center of the circle. The slope *ζ* of the line CH at the touch-down moment is perpendicular to the stance initial velocity vector and is calculated as

$$\zeta =\frac{{\dot{r}}_{0}\text{ sin }\theta_{0}+r_{0}{\dot{\theta }}_{0}\text{ cos }\theta_{0}}{{\dot{r}}_{0}\text{ cos }\theta_{0}-r_{0}{\dot{\theta }}_{0}\text{ sin }\theta_{0}}.$$ (6)

The length of the vertical line OC is

$$L=\zeta r_{0}\text{ sin }\theta_{0}+r_{0}\text{ cos }\theta_{0}.$$ (7)

The angle between the radial line CH and the vertical line OC is denoted by *θ*_1_ and the radius of the circle is obtained as

$$R=r_{0}\text{ sin }\theta_{0}\sqrt{1+\zeta^{2}}.$$ (8)

To calculate the necessary leg force we use a new polar coordinate system with the origin at C, radius of *R* and angle of *θ*_1_ as shown in Fig 3. The unit vectors of this coordinate system are denoted by $e_{r}{}_{{}_{1}}$ and $e_{\theta }{}_{{}_{1}}$. The dynamic equations of PMB in the new coordinate system are

$$\left\{ \begin{array}{l} \;F_{r}\text{ sin }(\theta_{1}+\theta )-mg\text{ sin }\theta_{1}=m(R{\ddot{\theta }}_{1}+2\dot{R}{\dot{\theta }}_{1})\\ -F_{r}\text{ cos }(\theta_{1}+\theta )+mg\text{ cos }\theta_{1}=m(\ddot{R}-R{\dot{\theta }}_{1}{}^{2}) \end{array}\right..$$ (9)

where *F*_*r*_ is the leg force and *mg* is the weight of the robot.

Because the radius *R* of the circle is constant during stance phase, its time derivatives will be zero in Eq (9). The necessary leg force is calculated using the second equation of Eq (9) as

$$F_{r}=\frac{mR{\dot{\theta }}_{1}{}^{2}+mg\text{ cos }\theta_{1}}{\text{cos}(\theta_{1}+\theta )}.$$ (10)

According to Fig 3 the angle *θ* is

$$\theta ={\text{tan}}^{-1}\left(\frac{R\text{ sin }\theta_{1}}{L-R\text{ cos }\theta_{1}}\right).$$ (11)

Substituting Eqs (10) and (11) into the first equation of Eq (9) results in an angular acceleration on the circular trajectory as

$${\ddot{\theta }}_{1}={\dot{\theta }}_{1}^{2}\text{ tan}\left(\theta_{1}+{\text{tan}}^{-1}\left(\frac{R\text{ sin }\theta_{1}}{L-R\text{ cos }\theta_{1}}\right)\right)+\left(\frac{g}{R}\right)\text{cos }\theta_{1}\text{ tan}\left(\theta_{1}+{\text{tan}}^{-1}\left(\frac{R\text{ sin }\theta_{1}}{L-R\text{ cos }\theta_{1}}\right)\right)-\left(\frac{g}{R}\right)\text{sin }\theta_{1}.$$ (12)

This nonlinear second order differential equation with respect to *θ*_1_ is solved numerically with the initial condition of

$$\left\{ \begin{array}{l} \theta_{1}\left(0\right)={\text{cot}}^{-1}\;\zeta ={\text{cot}}^{-1}\left(\frac{{\dot{r}}_{0}\text{ sin }\theta_{0}+r_{0}{\dot{\theta }}_{0}\text{ cos }\theta_{0}}{{\dot{r}}_{0}\text{ cos }\theta_{0}-r_{0}{\dot{\theta }}_{0}\text{ sin }\theta_{0}}\right)\\ {\dot{\theta }}_{1}\left(0\right)=-\frac{V_{0}}{R}=\frac{-{\dot{r}}_{0}\text{ cos }\theta_{0}+r_{0}{\dot{\theta }}_{0}\text{ sin }\theta_{0}}{r_{0}\text{ sin }\theta_{0}} \end{array}\right.$$ (13)

to obtain *θ*_1_ and ${\dot{\theta }}_{1}$ versus time. By substituting this solution into Eq (11) the necessary leg force profile is calculated numerically from Eq (10) to generate the circular-arc trajectory.

### Polynomial trajectory in stance phase

As a general framework we define the trajectory of the robot COM to be a polynomial of degree 2*n*. Since periodic running gaits of SLIP have symmetric stance trajectory, we consider symmetric polynomials with even degrees. The trajectory equation and its time derivatives are written as

$$y=\sum_{k=0}^{n}a_{k}x^{2k}$$ (14)

$$\dot{y}=\sum_{k=1}^{n}a_{k}(2k)\dot{x}x^{2k-1}$$ (15)

$$\ddot{y}=\sum_{k=1}^{n}\left(a_{k}\left(2k\right)\left(2k-1\right){\dot{x}}^{2}x^{2k-2}+a_{k}\left(2k\right)\ddot{x}x^{2k-1}\right)$$ (16)

in which parameters *a*_*k*_ can be chosen or designed using COM initial position, velocity and acceleration for stance phase. The Newtonian equations of motion in this phase are written using Eq (2) as

$$\left\{ \begin{array}{l} F_{r}.\frac{x}{\sqrt{x^{2}+y^{2}}}=m\ddot{x}\\ F_{r}.\frac{y}{\sqrt{x^{2}+y^{2}}}-mg=m\ddot{y} \end{array}\right..$$ (17)

Substituting *F*_*r*_ from Eq (16), *y* from Eq (14) and $\ddot{y}$ from Eq (16) into the first equation of Eq (17), the horizontal displacement differential equation is obtained as

$$\ddot{x}=\frac{gx+\sum_{k=1}^{n}a_{k}\left(2k\right)\left(2k-1\right){\dot{x}}^{2}x^{2k-1}}{a_{0}+\sum_{k=1}^{n}a_{k}\left(-2k+1\right)x^{2k}}.$$ (18)

The second order nonlinear differential Eq (18) should be solved numerically with the initial condition of

$$\left\{ \begin{array}{l} x\left(0\right)=-r_{0}\text{ cos }\theta_{0}\\ \dot{x}\left(0\right)=-{\dot{r}}_{0}\text{ cos }\theta_{0}+r_{0}{\dot{\theta }}_{0}\text{ sin }\theta_{0} \end{array}\right.$$ (19)

to obtain *x*, $\dot{x}$, $\ddot{x}$ versus time. Then *y* versus time is calculated using Eq (14) and substituting those into the first equation of Eq (17) results in the necessary leg force profile to generate the desired polynomial trajectory by COM in stance phase as

$$F_{r}=\frac{m\ddot{x}\sqrt{x^{2}+y^{2}}}{x}$$ (20)

### Verification of the proposed control law

To verify the proposed control laws to generate arbitrary trajectories for PMB in stance phase, we apply the calculated force profile *F*_*r*_ and the weight to the point mass, with the initial conditions, to generate a trajectory. This trajectory should be identical to the primarily desired circular-arc or polynomial trajectory. To do so, we use equations of motion in polar coordinate system shown in Fig 1b as

$$\left\{ \begin{array}{l} \;F_{r}-mg\text{ cos }\theta =m(\ddot{r}-r{\dot{\theta }}^{2})\;\\ mg\text{ sin }\theta =m(r\ddot{\theta }+2\dot{r}\dot{\theta }) \end{array}\right..$$ (21)

Eq (21) can be solved with known *F*_*r*_ and stance initial condition *r*_0_, *θ*_0_, ${\dot{r}}_{0}$, ${\dot{\theta }}_{0}$ to obtain generated trajectories by the proposed control laws in stance phase.

### Cost of transport

The cost of transport (COT) is an energy expenditure index that is used to evaluate biped waking and running efficiency. COT is defined as the energy exhausted by the motors per unit weight of the robot per unit distance traveled. To calculate the exhausted energy of motors, we use the absolute value of the product of the motor force and its displacement.

$$COT=\frac{W}{mgL}=\frac{1}{mgL}\int_{0}^{t_{CS}}\left|F_{r}\dot{r}\right|dt.$$ (22)

where *W* is the exhausted energy of the motors, *mg* is the total weight of the robot, *L* is the range of one running step on the ground, *t*_*CS*_ is the time duration of one complete step, *F*_*r*_ is the leg actuator force, and $\dot{r}$ is the leg length variation rate.

## Simulation Results

### SLIP model

**Fig 4:**
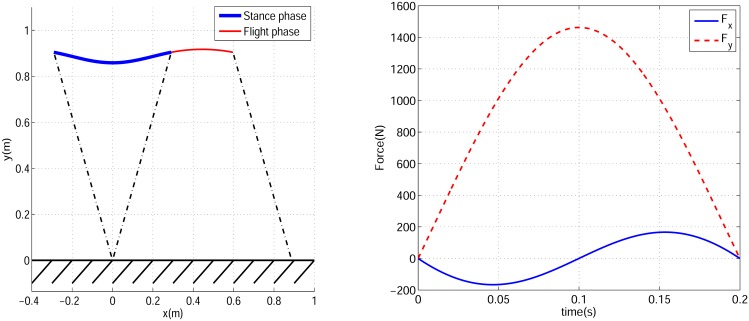
(a) SLIP model trajectory for one complete step of periodic running, (b) Horizontal and vertical components of toe force profiles.

A SLIP with a mass of 61.9 *kg*, a spring stiffness of 16 *kN*/*m* and a spring free length of 0.95 *m* is considered here to generate the running gait. These values are for the equivalent SLIP model for a typical biped robot named ATRIAS ‎[24]. Starting from an appropriate stance phase initial condition, the SLIP running dynamic Eq (3) can be solved to calculate stance and flight trajectories for one step, as well as the initial condition for stance phase of the next step. A SLIP running Poincare map is composed of dynamic equations of one complete step of running and maps the stance phase initial state vector of one step to the initial stance sate vector of the next step. For a given running velocity, a root finding problem can be solved to find the fixed point of this map which shows a stance phase initial condition that generates a periodic running gait. We solved this problem for the above-mentioned SLIP model with touch-down horizontal and vertical velocity components of *V*_*x*_ = 3 *m*/*s* and *V*_*y*_ = −0.5 *m*/*s* respectively. Its relevant fixed point becomes $x_{s}^{*}={\left[0.95,\;0.3112,\;-1.395,\;-2.845\right]}^{T}$. Then dynamic Eq (3) are solved with this initial condition that generates a periodic running gait with the desired velocity. Fig 4a shows a SLIP trajectory in stance phase and flight phase. In this figure, thick and thin solid lines show stance and flight trajectories respectively, and dash-dot lines show leg angles in touch-down and take-off events. Fig 4b shows GRF components for this gait in which solid and dashed lines stand for horizontal and vertical components respectively. This figure depicts a single-hump profile for vertical GRF and a sinusoidal shaped profile for horizontal GRF. The COM trajectory and GRF components in Fig 4 are qualitatively similar to human and animal running dynamic specifications ‎[25].

A PMB model is assumed to have the same mass and free leg length as the SLIP model. Then the proposed gait planning methods are applied to this model to generate various running gaits with circular and polynomial stance trajectories. All gaits have the same initial stance phase $x_{s_{0}}={\left[r_{0},\;\theta_{0},\;{\dot{r}}_{0},\;{\dot{\theta }}_{0}\right]}^{T}$ as SLIP gait and all have touch-down velocity components of *V*_*x*_ = 3 *m*/*s* and *V*_*y*_ = −0.5 *m*/*s*. Symmetric trajectory results in a mirrored take-off condition with the state vector of $x_{TO}={\left[r_{0},\;-\theta_{0},-\;{\dot{r}}_{0},\;{\dot{\theta }}_{0}\right]}^{T}$ and velocity components of *V*_*x*_ = 3 *m*/*s* and *V*_*y*_ = 0.5 *m*/*s*. So all generated gaits for PMB will have a fixed step length and fixed touch-down and take-off velocities.

### Circular-arc trajectory for PMB in stance phase

**Fig 5:**
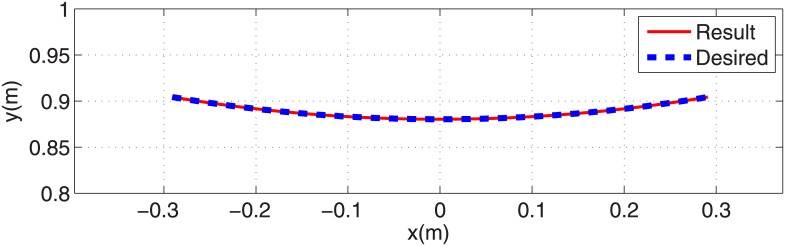
The desired and actual arc trajectories in stance phase.

Using the desired initial condition we perform the procedure described in section 3.1 step by step to calculate the necessary leg force to generate a circular-arc trajectory in stance phase. The desired trajectory for stance phase is found using L, R and *θ*_1_(0) from Eqs (7), (8) and (13). The necessary force profile for the leg actuator is calculated from Eq (9) and then is substituted into the differential equations in Eq (21). These equations are solved with the desired initial condition to calculate the actual trajectory in stance phase. The desired and resulting COM trajectory are exactly the same (Fig 5). This verifies our gait planning method.

**Fig 6:**
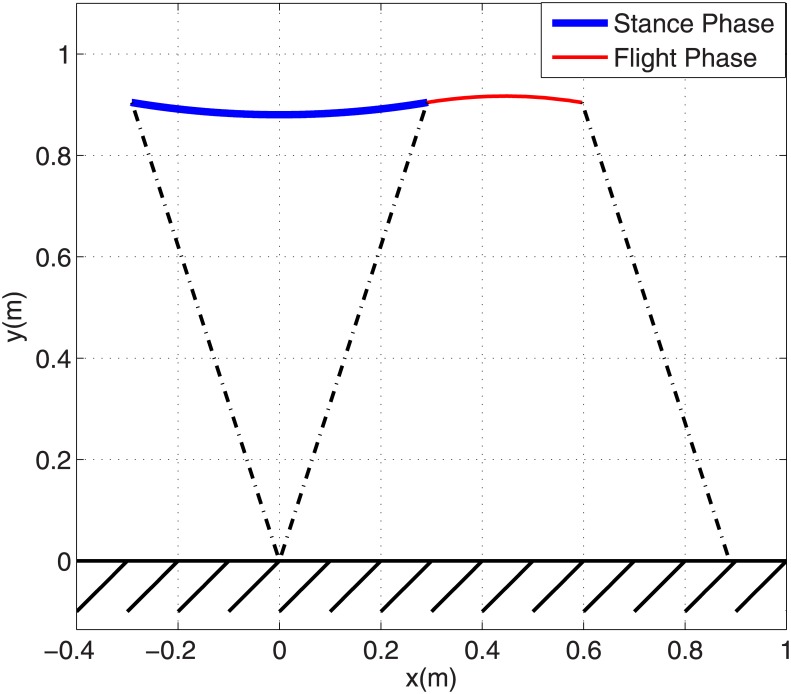
PMB trajectory in one complete step of periodic running with arc trajectory in stance phase.

**Fig 7:**
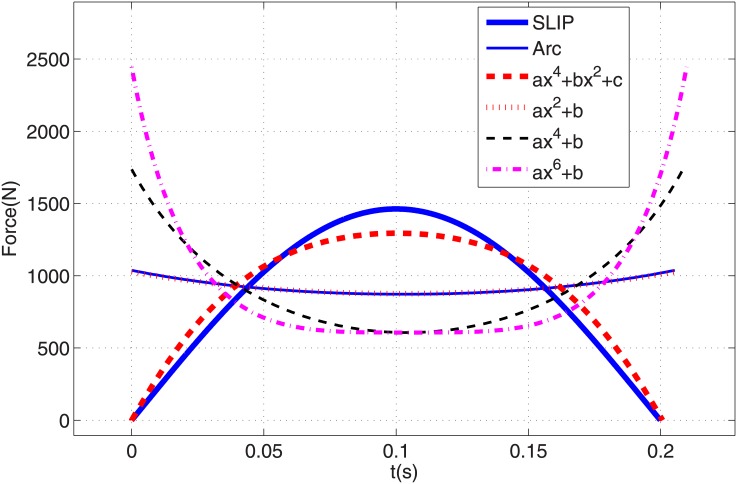
COM horizontal speeds during stance phase for SLIP and PMB models.

The COM trajectory for one complete running step is shown in Fig 6 in which the thick and thin solid lines stand for stance and flight trajectory respectively, and dash-dot lines show leg potion in touch-down and take-off events. It can be seen that the overall trajectory is similar to a SLIP trajectory. The resulting necessary force profile for the leg actuator is depicted in Fig 7 by a thin solid line and its parameters are shown in Table 1. The leg force starts at its maximum value of 1038 N and reaches its minimum value of 872 N in mid-stance. Note it has a different trend than the SLIP force profile which starts at zero and has maximum value of 1463 N in mid-stance. The maximum required leg force for a circular-arc trajectory is 29% less than with SILP, however it starts from its maximum value. Providing such a large force instantaneously just after touch-down is very difficult in practice.

**Table 1:** Running gait parameters for various trajectories in stance phase.

Path	Stance Start Leg Force (N)	Mid-Stance Leg Force (N)	Stance Time(s)	COT
Circular Arc	1038	872.2	0.2054	0.2463
*y* = *ax*^2^ + *b*	1024	876	0.2053	0.2463
*y* = *ax*^4^ + *b*	1737	607.1	0.2085	0.2440
*y* = *ax*^6^ + *b*	2445	607.1	0.2098	0.2432
*y* = *ax*^4^ + *bx*^2^ + *c*	0	1295	0.2007	0.2454
*y* = *ax*^6^ + *bx*^4^ + *cx*^2^ + *d*	0	Max = 1092.4	0.2024	0.2459
Force-actuated SLIP	0	1463	0.1999	0.2422

### Polynomial trajectories for PMB in stance phase

#### Polynomials with two parameters

**Fig 8:**
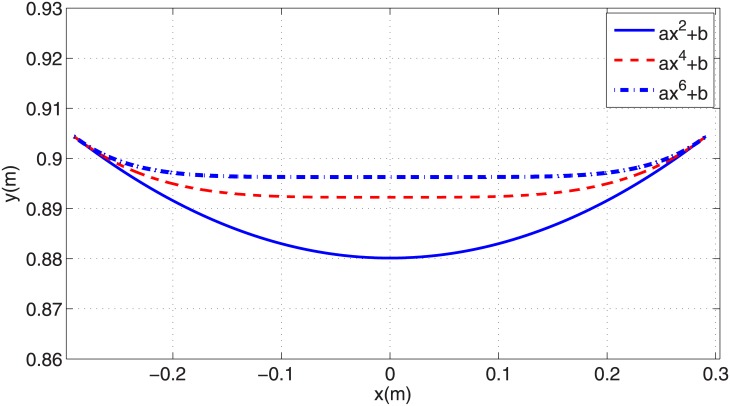
Generated polynomial trajectories of degrees 2, 4 and 6 with two parameters in stance phase.

In this section various polynomial trajectories of form Eq (14) are generated for the stance phase of PMB steady running. The first challenge is how to define the polynomial coefficients using the stance phase initial conditions. The stance phase initial state vector $x_{s_{0}}$ defines the COM initial position [*x*_0_, *y*_0_]^*T*^ and velocity ${\left[{\dot{x}}_{0},\;{\dot{y}}_{0}\right]}^{T}$. Substituting these points into Eqs (14) and (15) results in two algebraic linear equations with two unknown polynomial coefficients. So we consider even functions with only two coefficients: *y* = *ax*^2^ + *b*, *y* = *ax*^4^ + *b*, and *y* = *ax*^6^ + *b*. These paths with coefficients calculated from the stance phase initial state vector are shown in Fig 8. Using the procedure of Section 3.2 the required leg actuator force profile for each of these trajectories are calculated from Eq (20). Fig 7 and Table 1 show the resulting force profiles. All of them start and end at their maximum value, and reach their minimum value in mid-stance, similar to a circular-arc path. As the number of the degree of the polynomial increases, the maximum value of force grows and its minimum value decreases. The force profile corresponding to a degree-2 polynomial is very close to a circular trajectory force profile. The maximum force increases with polynomial degree. Because practical robots have motor torque restrictions, providing such large forces at the beginning of stance phase is not feasible.

#### Tuned degree-4 polynomial

**Fig 9:**
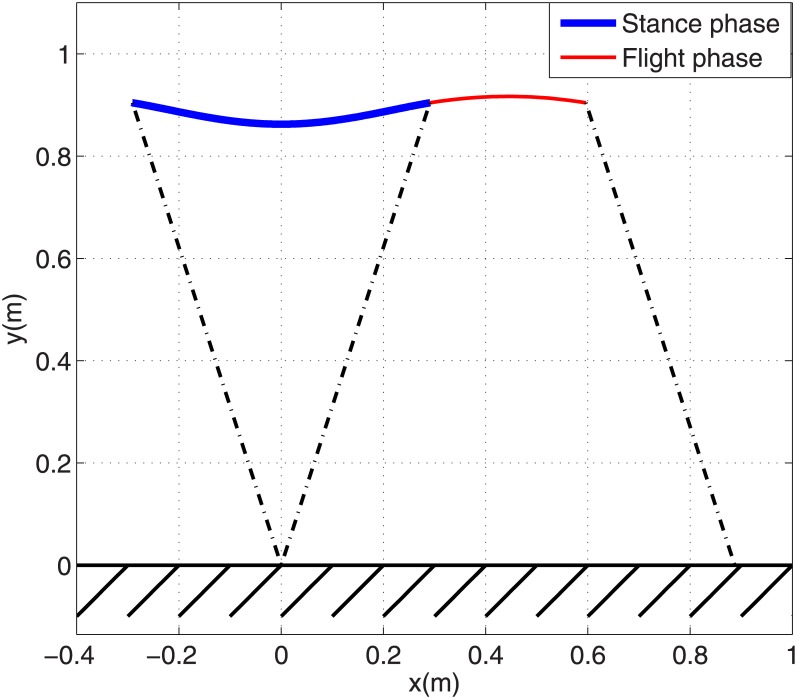
PMB model trajectory in one complete step of periodic running with tuned degree-4 polynomial trajectory in stance phase.

**Fig 10:**
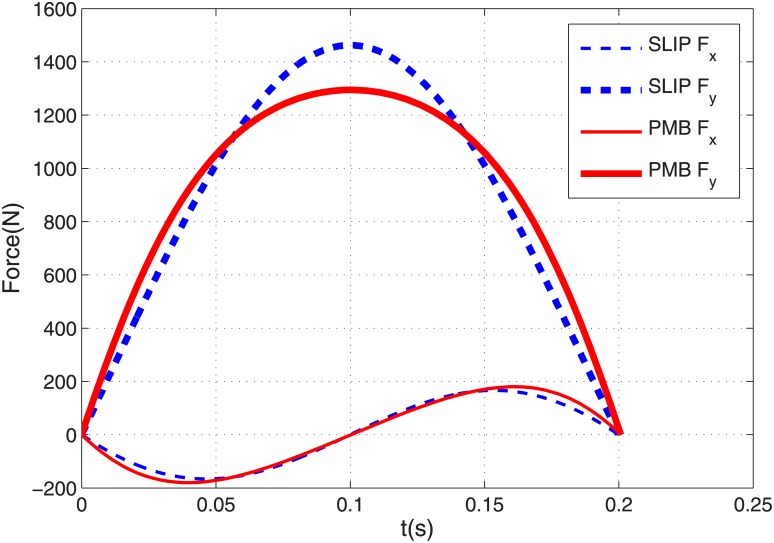
Horizontal and vertical components of toe force profiles for SLIP model and PMB model with tuned degree-4 trajectory in stance phase.

To make the leg force profile start and end at zero, one additional constraint is required in the stance phase initial condition. The acceleration of the body just before touch-down is ${\left[{\ddot{x}}_{0},{\ddot{y}}_{0}\right]}^{T}={\left[0,\;-g\right]}^{T}$, where *g* is the acceleration of gravity. To have the GRF start at zero, this acceleration constraint should be added to the stance phase initial condition. So in this case we have three points (position, velocity and acceleration) to be substituted into Eqs (14), (15) and (16). Therefore three coefficients of the path polynomial are required by these initial conditions, and we choose an even function of degree 4 for the polynomial *y* = *ax*^4^ + *bx*^2^ + *c*. The required leg force to make the robot undergo this trajectory during stance is calculated from Eq (20). Then the resulting force profile is substituted into differential Eq (21) to derive the actual stance trajectory. The desired and actual trajectories are verified to be coincident, similar to Fig 5. Fig 9 shows the PMB trajectory for one step of running using this tuned degree-4 polynomial. This trajectory is very similar to SLIP with the same parameters. The stance phase GRF components for both SLIP and PMB with a tuned degree-4 polynomial are shown in Fig 10. It is observed that these two models have almost the same horizontal GRF components but the vertical component of SLIP is sharper, *i*.*e*. the PMB model requires less maximum motor torque and less ground friction coefficient than SLIP. These are the main advantages of PMB with a tuned degree-4 polynomial compared to SLIP running. Note also that physical implementation SLIP-based robots, such as ATRIAS ‎[20], have motors in series to the spring.

**Fig 11:**
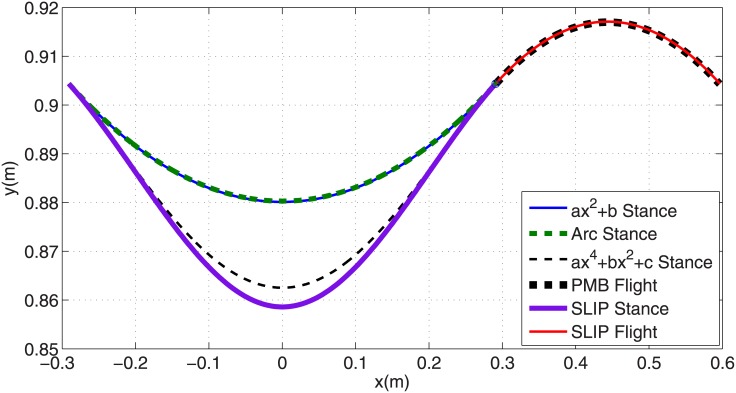
Comparison of various generated trajectories for SLIP and PMB models in stance and flight phases.

The COM trajectories for one step of running for the all previously generated gaits are shown in Fig 11. All of these gaits have the same touch-down and take-off configuration but different stance trajectories. The flight phases of all these gaits are coincident. In stance phase, it is noticeable that the circular arc and degree-2 polynomial trajectories are very close to each other and the tuned degree-4 polynomial is relatively close to the SLIP trajectory. Horizontal components of COM velocities for these trajectories during stance phase are shown in Fig 7. It is noticeable that again the velocity profiles and the time duration of the circular-arc and degree-2 polynomial trajectories are almost coincident, and the velocity profiles and time duration of SLIP model and degree-4 polynomial trajectories are also close to each other. All of these trajectories start and end with the same velocity of *V*_*x*_ = 3 *m*/*s*, but their velocities differ slightly during stance phase. Because the start and end points are fixed, this causes different stance times for these trajectories. The SLIP model has a horizontal velocity of 2.83 *m/s* in mid-stance and the degree-2 polynomial trajectory has 2.75 *m/s*. The stance phase duration is 0.200 *s* for the SLIP model, 0.201 *s* for the degree-4 trajectory, and 0.205 *s* for the degree-2 and circular-arc trajectories. All of the trajectories have a flight phase duration of 0.102 *s* and a complete step stride of 0.888 *m*. So the average running speed is 2.94 *m*/*s* for the SLIP model, 2.93 *m*/*s* for the degree-4 trajectory (0.3% less than SLIP), and 2.89 *m*/*s* for the degree-2 trajectory (1.7% less than SLIP). Note all of the generated gaits in this paper use the same initial and final position/velocity for stance phase, and different trajectories differ less than 2% in average velocity.

**Fig 12:**
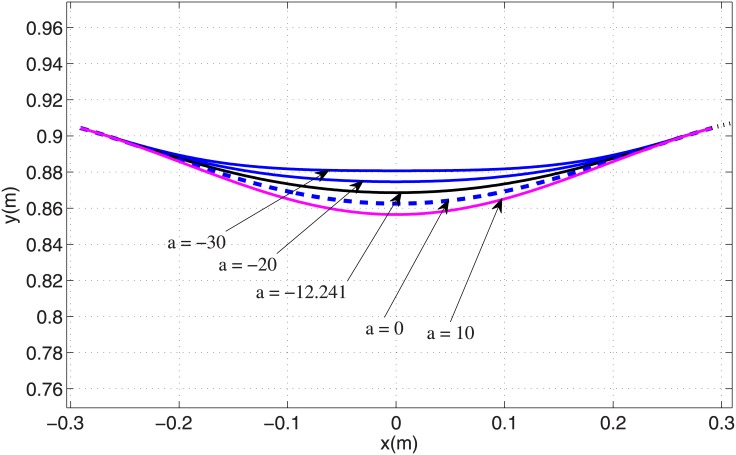
Needed leg force profiles vs time for various trajectories in stance phase.

The corresponding leg force profiles for the various trajectories are shown in Fig 12. It is desirable for the leg actuator force in stance phase to have an initial and final value of zero, and a peak value as small as possible. By this criteria, the tuned degree-4 polynomial has the best force profile specifications among these trajectories, with 11% less maximum force than SLIP. The smallest maximum leg force is found with the degree-2 polynomial, and it is 30% less than SLIP. Although this gait needs less motor force, it has the disadvantage of starting and ending at its maximum value. The COTs of the generated gaits are compared in Table 1. Notice that SLIP is a passive model with a COT of zero. In order to gauge whether the PMB gaits are energy efficient, we compare them to a SLIP model where the leg spring has been replaced by a force actuator that needs to generate the same force profile and displacement *i*.*e*. a force-actuated SLIP. The resulting force-actuated SLIP has the best COT with a value of 0.2422, but the PMB gaits have very similar COTs (Table 1). The worst PMB COTs are found with the degree-2 polynomial and the circular-arc trajectories, both 1.7% higher than force-actuated SLIP. The tuned degree-4 polynomial PMB has a COT only 1.3% higher.

#### Optimized degree 6 polynomial

**Fig 13:**
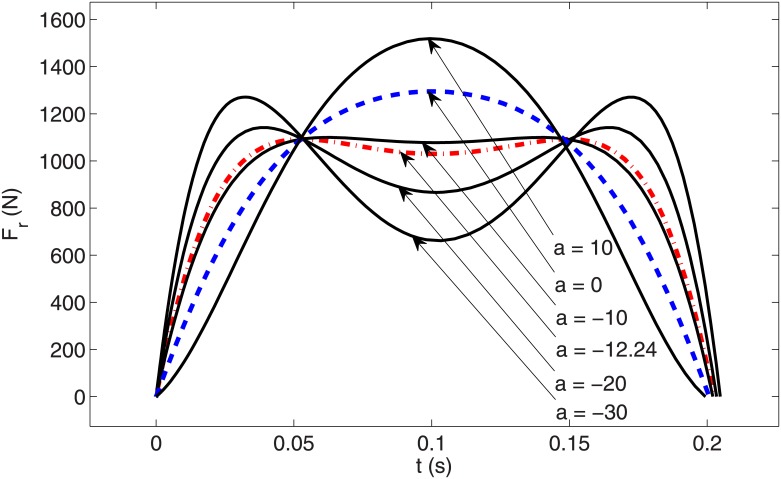
Degree 6 polynomial trajectories in stance phase with various coefficient *'a'*.

**Fig 14:**
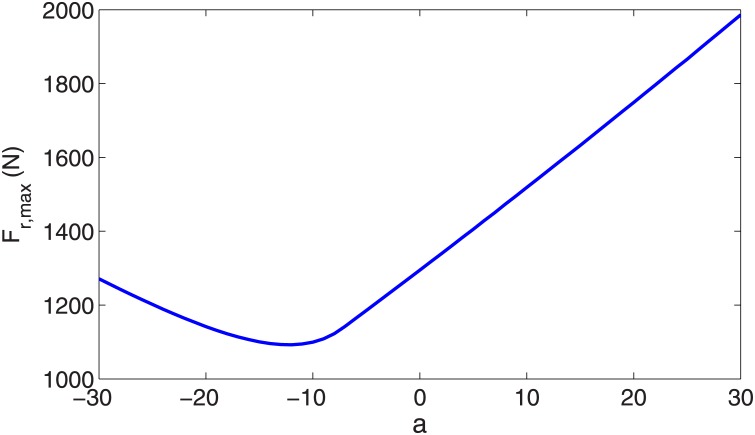
Needed leg force profiles vs time for degree 6 polynomial trajectories in stance phase with various values of coefficient *'a'*.

**Fig 15:**
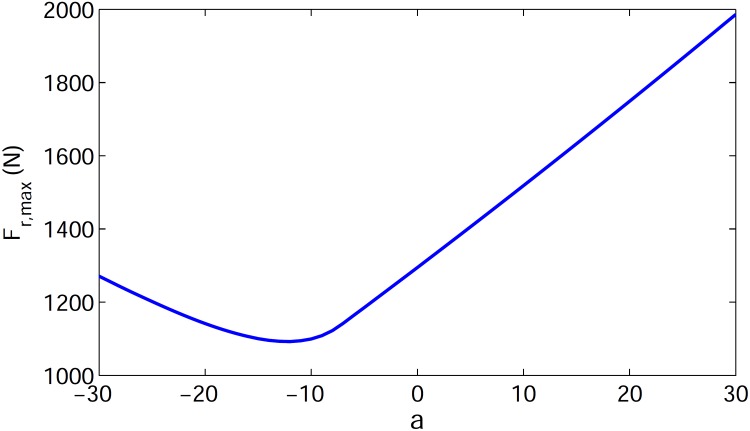
Variation of max leg force of stance phase vs. coefficient *'a'* for degree 6 polynomial trajectories.

We conclude that the PMB tuned degree-4 polynomial trajectory meets the main requirements of biped running. In an effort to discover more about optimal gaits, we next use an even function of degree 6 polynomial *y* = *ax*^6^ + *bx*^4^ + *cx*^2^ + *d*, where the coefficient *a* is a free parameter that satisfies the three initial conditions (similar to the tuned degree-4 polynomial). The relevant trajectories with coefficient *a* from -30 to 10 are shown in Fig 13, which illustrates that the leg length shortens with increasing *a*. The corresponding leg force profiles are shown in Fig 14, which depicts single-hump profiles for *a* > −7.22 and double-hump profiles for *a* < −7.22. (Note the case *a* = 0 in Fig 14 is identical to the tuned degree-4 polynomial in the previous section). This is an interesting result because a single-hump force profile is known as a characteristics of bipedal running gaits, while a double hump is characteristic of walking ‎[15,‎^16^,‎^25^]. The double-hump force profile for a running gait is a new result to the best of our knowledge. It is likely that one could capture running gaits with triple and quadruple-hump force profiles by choosing higher-order polynomial trajectories. We claim that a single-hump force profile is not a characteristics of bipedal running gaits (at least in the field of robotics). Although all observed biologic running gaits demonstrate single-hump force profiles, either single or double-hump profiles can be chosen for the running gaits of robots according to engineering requirements. One important restriction for the running gaits of robots is the maximum torque of the motors. To overcome this restriction we need gaits with less maximum leg force. It can be observed in Fig 14 that there is an optimum value for the coefficient *a* that minimizes maximum leg force, resulting in a double-hump profile. The variation of maximum leg force with respect to *a* is shown in Fig 15, which shows that the optimum value of the free parameter is *a* = −12.24. The leg force profile corresponding to the optimal value of coefficient *a* is shown in Fig 14 by a dash-dot line.

The important specifications of the force profiles of all previously mentioned stance trajectories are summarized in Table 1. The row for the degree-6 polynomial in this table uses the optimal value *a* = −12.24 and for mid-stance leg force we use the maximum leg force in the double-hump profile. Notice that stance times differ a bit for the various trajectories because we constrained only stance starting velocities, ending velocities, and its path profile (not average running speed). These differences are not significant and do not cause any problems to our investigation.

## Effects of Stance Initial Condition on Running Efficiency

**Fig 16:**
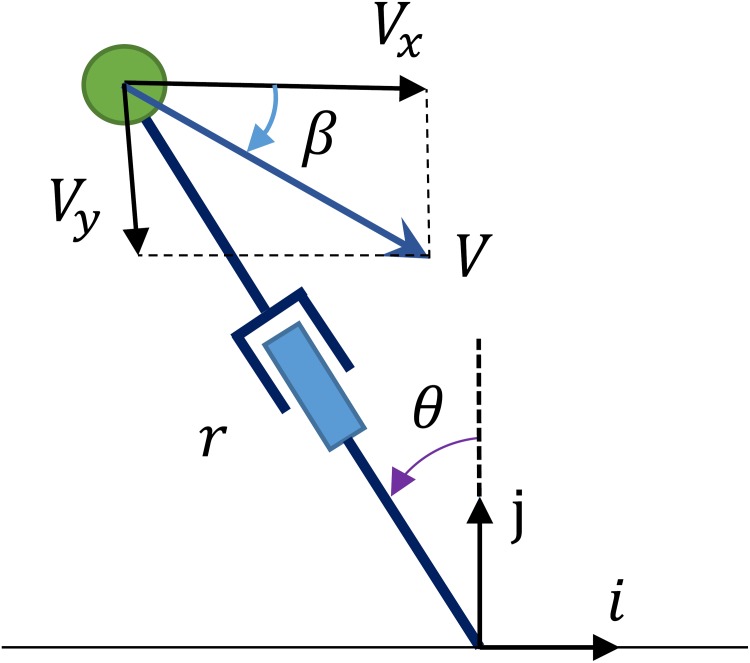
State variables of PMB in stance phase.

We defined the stance phase state vector as $x_{s}=\left[r,\;\theta ,\;\dot{r},\;\dot{\theta }\right]$. Equivalently the velocity can be defined by *V*_*x*_, *V*_*y*_, where the vertical velocity can be written as *V*_*y*_ = *V*_*x*_ tan *β* according to Fig 16, *β* is the angle of velocity vector *V* with respect to the horizontal, and *θ* is the leg angle with respect to the vertical. So instead we can use [*r*, *θ*, *V*_*x*_, *β*] as the stance phase state vector and [*r*_0_, *θ*_0_, *V*_*x*0_, *β*_0_] as the stance initial condition. In this section we examine the effects of attack angle *θ*_0_ and velocity angle *β*_0_ on the efficiency of the running of a biped robot with free leg length of *r*_0_ and touch-down speed of *V*_*x*0_. Thus *r*_0_, *V*_*x*0_ are assumed to be constant with values of 0.95m and 3m/s respectively.

**Fig 17:**
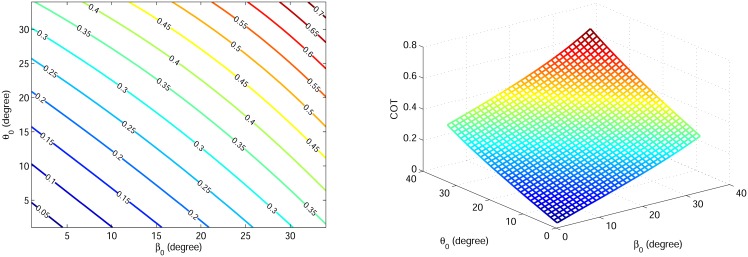
(a) Contour plot and (b) 3D plot of COT variation relative to *θ*_0_ and *β*_0_.

**Fig 18:**
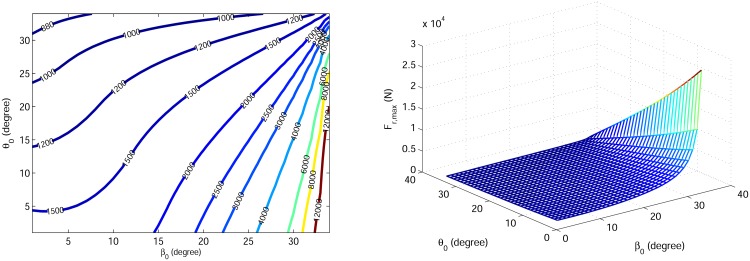
(a) Contour plot and (b) 3D plot of maximum leg force variation relative to *θ*_0_ and *β*_0_.

Because the tuned degree-4 polynomial trajectory includes the necessary biped running dynamics and since we aim to investigate trends of gait specifications, we choose the tuned degree-4 polynomial to generate gaits in this section. For each initial condition [*r*_0_, *θ*_0_, *V*_*x*0_, *β*_0_] there is a unique tuned degree-4 polynomial. Parameters *θ*_0_, *β*_0_ are varied from 1° to 34° to generate feasible running gaits. Using the proposed control strategy, the stance trajectory is uniquely defined for each initial condition. The COT and the maximum leg force are calculated in these cases, shown in Figs 17 and 18. It turns out that the COT increases with both *θ*_0_ and *β*_0_, and it approaches zero when both *θ*_0_ and *β*_0_ approach zero. Also it can be seen that maximum leg force decreases with *θ*_0_ and increases with *β*_0_.

**Fig 19:**
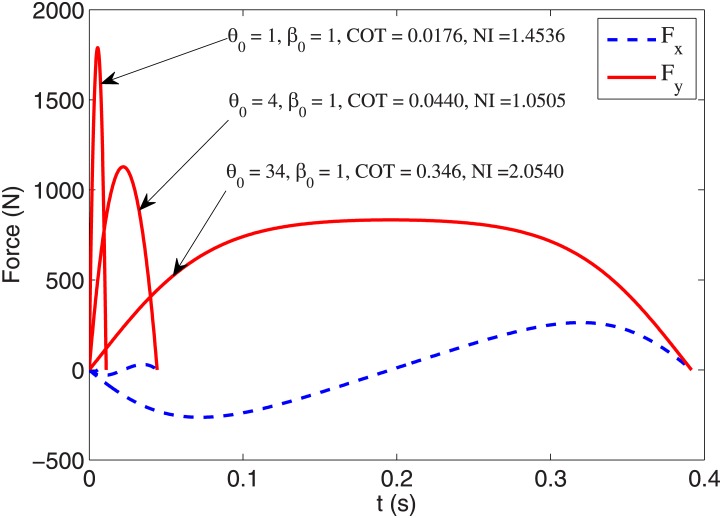
Horizontal and vertical components of toe force profiles with optimal values of stance initial condition.

Optimal values of stance initial condition within the intervals are summarized in Table 2. The best COT, with value 0.0176, is obtained by the smallest attack angle and velocity angle (*θ*_0_ = 1°, *β*_0_ = 1°), but this produces a relatively large leg force *F*_*r*,*max*_ = 1789.8 *N*. The smallest maximum leg force is obtained by the smallest velocity angle *β*_0_ = 1° and the largest attack angle *θ*_0_ = 34°, but it results in a COT = 0.346, which is a large value relative to the other gaits shown in Table 1. To find an overall optimum gait considering the trade-off between COT and maximum leg force, we define a normalized index as

$$NI=\frac{COT}{0.2454}+\frac{F_{r,\text{max}}}{1295}$$ (23)

which is normalized relative to the COT and *F*_*r*,*max*_ values of the tuned degree-4 polynomial trajectory gait. Minimizing this normalized index generates a gait with *θ*_0_ = 4°, *β*_0_ = 1 that results in COT = 0.0440 and *F*_*r*,*max*_ = 1228.2 *N*. Profiles of leg force components *F*_*x*_, *F*_*y*_ for the gaits with minimum COT, *F*_*r*,*max*_ and *NI* are shown in Fig 19. It is worth noticing that all these gaits have the same horizontal velocity but considerably different time duration and step length. This implies that shorter gaits will need a higher frequency of running steps to travel the same horizontal distance in the same amount of time.

**Table 2:** Optimal values of stance initial condition.

	*θ*_0_ = 1°, *β*_0_ = 1	*θ*_0_ = 34°, *β*_0_ = 1°	*θ*_0_ = 4°, *β*_0_ = 1
COT	0.0176	0.346	0.0440
*F*_*r*,*max*_ (N)	1789.8	833.4	1128.2
Normalized Index	1.4536	2.0540	1.0505

Here we put only the normalized sum of COT and *F*_*r*,*max*_ as the objective function to obtain an optimal gait. However, humans, animals and robots have some more constraints which should be taken into account when choosing an appropriate gait. For example choosing a relatively small attack angle *θ*_0_ = 4° causes an optimal COT and *F*_*r*,*max*_, but this requires a high frequency of feet switching that would cause muscle fatigue in humans/animals and challenges in robots due to motor response-time restrictions. Thus choosing a larger attack angle makes more physical sense.

Choosing velocity angle *β*_0_ as small as possible is beneficial in minimizing both COT and *F*_*r*,*max*_. This is convenient because it has been observed that COM travels on an (almost) horizontal line during high-speed, high-efficiency running in both humans and animals.

## Conclusions

A point mass biped (PMB) model provided the basis for generating arbitrary symmetric trajectories in the stance phase of steady running. To show the generality of the method, a circular-arc trajectory and polynomials of various degrees (all satisfying the initial stance conditions) constituted desired trajectories in stance. The algorithm calculated the required force profile for the leg actuator for each trajectory. We found that paths from polynomials with only two parameters (satisfying initial position and velocity) do not start and end with zero force, which is not desirable. To overcome this problem we added the stance phase initial acceleration to the initial condition, then considered paths with three and four parameters. A degree-4 polynomial with three parameters resulted in a path and force profile very similar to those from a SLIP model, with nearly-equal COT and a maximum leg force 11% less. For a degree-6 polynomial with four parameters, optimizing the maximum leg force resulted in a gait with maximum leg force of 25% less than SLIP. The degree-6 polynomial trajectory can generate biped running gaits with either single-hump or double-hump GRF (previously double-hump profiles was counted as a characteristics of walking ‎[25]). It is worth mentioning that for a defined touch-down velocity, a SLIP model needs a unique attack angle to generate a periodic running gait. However, PMB can generate periodic running gaits with arbitrary touch-down velocities and attack angles (within reasonable limits). We then investigated the effects of attack angle and velocity angle on running efficiency. Choosing the velocity angle as small as possible reduced both COT and maximum leg force. Larger attack angles increased COT and reduced maximum leg force, so there is an optimum value. Using PMB instead of SLIP as a template for multibody biped robots offers some practical advantages: a PMB trajectory can be optimized to overcome real-world limitations such as maximum motor torque, ground coefficient of friction, etc.

We generated symmetric trajectories for the stance phase in this work. Because this was analytic, exact trajectory planning did not deal with stability issues. Implementing these open-loop gaits on consecutive running steps of an underactuated robot will generate periodic unstable gaits. Designing controllers to stabilize these planned underactuated gaits (for example using Poincare map control ‎[26] or energy level control ‎[24]) defines future research work. Also, in future work asymmetric trajectories can be investigated in order to reject disturbances in the stance initial condition for steady running, transient running gaits, or running on stairs. Furthermore, this method of gait planning can be applied to 3D PMB and biped robot models with higher degrees of freedom.
